# Association of lower vitamin a levels in neonates and their mothers with increased risk of neonatal late-onset sepsis: A case-control study

**DOI:** 10.34763/jmotherandchild.20222601.d-22-00023

**Published:** 2023-02-22

**Authors:** Farhad Abolhasan Choobdar, Maral Ghassemzadeh, Fatemeh Aslanbeigi, Mohammad Attarian, Leila Robatmeili, Hanie Rahimian, Behzad Haghighi Aski, Ali Manafi Anari

**Affiliations:** Department of Pediatrics, School of Medicine, Hazrat e Ali Asghar Pediatric Hospital, Iran University of Medical Sciences, Tehran, Iran; Dr.Shariati general Hospital, Tehran University of Medical Sciences, Jalal Al-Ahmad Ave, Tehran, Iran; Kashan University of Medical Sciences, Kashan, Iran; Firoozgar General Hospital related to Iran University of Medical Sciences Kashan, Iran; Department of Pediatrics, School of Medicine, Hazrat e Ali Asghar Pediatric Hospital, Iran University of Medical Sciences, Kashan, Iran

**Keywords:** Neonate, Vitamin A, Sepsis

## Abstract

**Background:**

In developing countries, neonatal sepsis is one of the major causes of mortality and morbidity. Vitamin A deficiency also affects the immune system severely and is associated with various neonatal infections. We aimed to compare maternal and neonatal vitamin A levels among neonates with and without late-onset sepsis.

**Material and methods:**

40 eligible infants were entered into this case-control study according to inclusion criteria. The case group included 20 term or near-term infants who had late-onset neonatal sepsis from three to seven days of life. The control group consisted of 20 term or near-term infants who were icteric hospitalized neonates without sepsis. Demographic, clinical and paraclinical features, as well as neonatal and maternal vitamin A levels, were compared between the two groups.

**Results:**

The average gestational age of the neonates was 37.1 ± 1.2, ranging from 35 to 39 days. There was a significant difference between the septic and non-septic groups in terms of white blood cell and neutrophil count, C-reactive protein, and neonatal and maternal vitamin A levels. A Spearman correlation analysis showed a significant direct correlation among maternal and neonatal vitamin A levels (correlation coefficient = 0.507; P-value = 0.001). Multivariate regression analysis showed that neonates’ vitamin A level had a significant direct association with sepsis (OR: 0.541; P-value=0.017).

**Conclusion:**

Our findings demonstrated the association of lower vitamin A levels in neonates and their mothers with an increased risk of late-onset sepsis, which emphasizes the importance of the consideration of vitamin A level evaluation and its appropriate neonatal and maternal supplementation.

## Introduction

Neonatal sepsis is defined as a systemic inflammatory response syndrome subsequent to infection in the first 28 days of life. Sepsis is confirmed on the basis of microbiological cultures or strong clinical evidence in favor of infection [1, 2]. In developing countries, neonatal sepsis is one of the major causes of mortality and morbidity in infants. As many as 1.6 million infant deaths are reported annually due to infections, mainly diagnosed as meningitis and sepsis. Neonatal infections account for 44% of all deaths in developing countries. Infants are more susceptible to invasive bacterial infections than other age groups; despite the lack of specific symptoms of sepsis in infants, the risk of developing and spreading sepsis is higher in this group [3, 4, 5, 6].

Reducing neonatal sepsis mortality is one of the most important public health concerns in developing societies. Various studies have shown that early diagnosis of sepsis based on its signs and symptoms may improve outcomes significantly [7, 8].

Vitamin A and its derivatives are all organic, unsaturated, fat-soluble, heat-sensitive compounds that play a fundamental role in embryonic development, growth, vision, reproduction, and immunity [9, 10, 11, 12]. Especially in infants and children, adequate amounts of this vitamin are necessary to support rapid growth and resistance to infections. Vitamin A is an anti-infective vitamin [13]. In addition to retinopathy, which is one of the effects of vitamin A deficiency, numerous studies report serious complications of infection due to vitamin A deficiency in children. Vitamin A deficiency affects the immune system on several levels, including the destruction of the integrity of the mucosal epithelial membrane, which acts as a protective barrier in the gastrointestinal, respiratory and urinary systems. It causes metaplasia and destruction of the defense mechanism of the squamous layer of the respiratory epithelium and microbial invasion [13]. Vitamin A deficiency also leads to a weakened immunity through dysfunction of macrophages and natural killer cells, monocytes, neutrophils and dendritic cells [14, 15]. It also increases the severity of enterovirus infections by reducing interferon alpha concentration and specific IgM concentration [16, 17]. Administration of this vitamin supplement in malnourished communities, in childhood, and during pregnancy reduces mortality due to infections [18].

Since vitamin A deficiency is a major issue in Iran [19, 20], we designed this comparative study to evaluate the influence of vitamin A level between neonates with and without late-onset sepsis (LOS).

## 2 Material and Methods

### 2.1 Study Settings

This study was a case-control study over a period of six months in the neonatal wards and neonatal intensive care units (NICUs). After obtaining informed consent from the parents of the infants, and ethics committee approval, 40 eligible infants were entered into the study according to the inclusion criteria. The case group included 20 term or near-term infants who had been hospitalized with late-onset neonatal sepsis diagnosis from three to seven days of age, while the control group consisted of 20 term or near-term icteric hospitalized neonates without any features of sepsis.

We treated all LOS patients with antibiotics according to our NICU protocol and existing literature. In the control group, we only used phototherapy for all patients with uncomplicated neonatal jaundice, without any medication [4].

### 2.2 Study Population

This study was performed on two groups: the case group included full-term and near-term infants (above 35 weeks) with sepsis who had been hospitalized due to late-onset neonatal sepsis between the third and seventh day of life and had not yet received any vitamin supplements. LOS was diagnosed based on clinical and paraclinical signs. We did a complete sepsis work-up for each neonate in both groups which included CBC diff, CRP, and blood cultures to rule out sepsis, and when necessary, we also took CSF cultures in septic group neonates. Moreover, in both groups, we also took urine cultures, because a UTI can be one of the important causes of both neonatal prolonged jaundice and late-onset sepsis. We consider prolonged jaundice to be a jaundice lasting more than 2 weeks; regardless, we took a urine culture to rule out late-onset sepsis in the control group or identify it in the case (sepsis) group. In septic neonates without any positive culture, we used Rodwell criteria as a precise instrument for sepsis diagnosis [4, 8, 47].

The control group consisted of 20 term or near-term infants who had been diagnosed with uncomplicated jaundice for any reason, provided that their bilirubin level was not close to the blood transfusion level, and who had been hospitalized for jaundice within three to seven days of their life, matching the case group. The neonates in control group had no suspected clinical or laboratory symptoms of sepsis, and their clinical and paraclinical signs, as well as CBC diff, CRP, B/C and urine culture, were not in favor of infection in any way, so these signs and symptoms strongly and definitely ruled out the presence of sepsis. It should be noted that with the exception of jaundice, the control group infants were found to be be completely healthy in other respects, having no other health problems, and they should not have received vitamin A supplements [21].

Study exclusion criteria among the neonates in both case and control group consisted of having inherited metabolic disorders, IUGR infants, infants with asphyxia, malabsorption syndromes, major anomalies, genetic and immunodeficiency diseases, and malignant underlying diseases, as well as infants who had received any form of vitamin A supplementation during their pre-hospitalization period (which means the first week of their life). Infants whose parents did not consent to their participation in the study, and those who died during hospitalization or were discharged from the hospital with personal consent before completing the course of treatment, were also excluded [12, 13, 22].

The reason we did not consider early-onset sepsis was that in the first three days of life, there may be many other transient and non-septic conditions which may mimic sepsis; for example, TTN, or transient tachypnea of neonates, or pneumonia may be clinically mistaken as sepsis. These conditions (especially TTN) which are even more common than infection, if not complicated, are mostly recovered after the third day of life. As such, we limited the inclusion criteria to late-onset sepsis to reduce the overlap with transient and non-septic conditions in the first days of life.

Neonates in each group were selected among eligible patients which fulfilled the selection criteria. The two groups were matched in terms of gestational age, birth weight, day of admission, age, feeding and delivery type, maternal age, and Apgar score, to minimize the effect of confounding factors.

### 2.3 Data collection

The case-to-case ratio in this study was one-to-one, and the main dependent variable examined was the level of vitamin A in maternal and neonatal serum. For infants in both groups, vitamin A levels were measured after the third day of life. Demographic data for each of the 40 infants, including Apgar score, gender, age, and weight based on the SECA 345 digital scale with a measurement accuracy of 3 grams, were collected.

### Laboratory Measurements

Maternal and neonatal vitamin A levels in both groups were measured by HPLC during the first week after delivery in the Gholhak clinical laboratory, by the Agilent G6410B Triple Quadrupole LC/MS system, which is a liquid chromatograph triple quadruple mass spectrometer that performs MS/ MS using three sets of parallel rods. The Agilent G6410-B Triple Quadrupole LC/MS system features a mass range of 15 to 1650 m/z (mass to charge ratio), with mass accuracy of < 0.1 u over 24 hours with MS/MS sensitivity of 0.5 pg (pictogram = 0.000000000001 grams) of reserpine inject on column. The results were recorded and stored blindly, through codes, for each infant. 2 to 3 cc of blood were taken in a tube without anticoagulation and after complete blood clotting, the serum was separated through a centrifuge, poured in a separate tube, and then kept at -20°. Due to the sensitivity of vitamin A to light and heat, the sampling was performed in special containers and sent to the reference laboratory as soon as possible. The normal range of vitamin A levels, according to the Gholhak clinical laboratory, is 30-80 microgram/dl (μg/dl).

According to the WHO, vitamin A deficiency in any age is defined as vitamin A level less than 21 μg/dl. However, many references consider 30 μg/dl as the cutoff point of vitamin A deficiency in human being of any age, because when the level of vitamin A reaches 30 μg/dl, the hepatic storage of vitamin A is greatly affected, and the immunity system as well as tissue repair mechanisms and respiratory epithelium begin to be disturbed, leading to the patient being very prone to sepsis even though they may not have obvious clinical symptoms. According to studies done in developed countries, vitamin A concentration in cord blood immediately after the birth of normal baby is 30 μg/dl; this amount is 18-24 μg/dl in developing countries. Considering all of the above, we defined vitamin A deficiency as vitamin A level less than 30 μg/dl [12, 13, 18, 22, 46].

### 2.4 Statistical analysis

The sample size is defined based on a study by Zhang et al [12], in which according to similar studies, the incidence of vitamin A deficiency was reported to be 58% in the sepsis group and 12% in the control group. Therefore, considering α=0.05, β=0.18, Z1-α/2= 1.96, Z1- B = 0.9, and also estimating a 10% drop out rate, the final sample size was calculated to be about 20 patients in each group.

Demographic data and maternal and neonatal serum levels of vitamin A in both sepsis and control groups were evaluated and analyzed with SPSS software version 25. Categorical variables were described as frequency rates and percentages, while continuous variables were described using mean and standard deviation (SD). Proportions for categorical variables were compared using the χ2 test, and the Fisher exact test was used when the data were limited. An independent sample t-test was used to compare the average level of vitamin A in the case and control groups. The chi-square test was utilized to check the compatibility of variables such as Apgar and gender in the two groups. A correlation test was used to evaluate the relationship between vitamin A levels in mothers and neonates. To evaluate the relationship between sepsis and neonatal or maternal factors, linear regression and logistic regression were used. A P-value of lower than 0.05 was considered significant.

### Ethical approval

No extra blood samples were taken from neonates; vitamin A levels were checked along with the blood that was sent for routine testing. Parents in both case and control groups were given the necessary information to obtain informed consent. This information included sampling and the amount of blood needed to test the level of vitamin A in mother and infant. If the parents agreed to their infant entering the study, this consent was recorded in written forms. All protocols were approved by the Ethics Committee of the Iran University of Medical Sciences. This study was carried out in compliance with the relevant guidelines. The Ethics Committee Approval code for this research is IR.IUMS.FMD.REC.1399.576. We ensure the quality and integrity of our research. We obtained informed consent from the parents of all infants participating in our study and also informed consent from the mothers who participated in our study. We respect the confidentiality and anonymity of our research respondents. We guarantee that the infants participated in the study voluntarily and according to their parents’ wishes. We also avoided any harm to our participants. There is no conflict of interest in this study and we did not have any financial support for this research.

## 3 Results

40 neonates were assembled as the case and control groups, including neonates with sepsis and non-septic neonates. The average gestational age of the neonates was 37.1 ± 1.2, ranging from 35 to 39 weeks. These neonates in septic and non-septic groups were matched according to their day of hospital admission, maternal age, gestational age, birth weight, feeding type and delivery type. These neonates were admitted to the hospital on about the 4^th^ day of their life. The clinical and demographical features of the participants in our study are demonstrated in Table 1. As demonstrated, there was a significant difference among the septic and non-septic group in terms of white blood cell (WBC) count, neutrophil count, C-reactive protein (CRP), vitamin A level, and maternal vitamin A levels. Also, the rate of sepsis was significantly higher in neonates who were born prematurely because of premature rupture of membrane (PROM) rather than premature labor pain or Preeclampsia (Table 1).

**Table 1:** Comparison of demographic and clinical features of neonates and their mothers among the septic and non-septic group

Variable		Total; *N* = 40	Sepsis; *n* = 20	Non-septic; *n* = 20	P-value*
Neonate age (days); mean±SD (range)		4.7 ± 1.3 (3 – 7)	4.9 ± 1.3 (3 – 7)	4.5 ± 1.3 (3 – 7)	0.397
Mother age (years); mean±SD (range)		28.2 ± 4.5 (19 – 36)	27.9 ± 4.6 (19 – 34)	28.6 ± 4.3 (21 – 36)	0.625
Birth weight	Average; mean±SD (range)	2717.0 ± 449.8 (1980 – 3600)	2850.0 ± 417.5 (2150 – 3600)	2584.0 ± 451.5 (1980 – 3600)	0.061
(grams)	<2500; n (%)	13 (32.5)	4 (20.0)	9 (45.0)	
	≥ 2500; n (%)	27 (67.5)	16 (80.0)	11 (55.0)	0.091
	Male	21 (52.5)	6 (30.0)	15 (75)	
Gender; n (%)	Female	19 (47.5)	14 (70.0)	5 (25.0)	**0.004**
	Average mean±SD (years); (range)	37.1 ± 1.2 (35 – 39)	37.4 ± 1.0 (35.4 – 39.0)	36.9 ± 1.3 (35 – 39)	0.205
Gestational age	Term; n (%)	27 (67.5)	15 (75.0)	12 (60.0)	
	Preterm; n (%)	13 (32.5)	5 (25.0)	8 (40.0)	0.311
Cause of	Preterm labor pain	8 (20.0)	0 (0)	8 (40.0)	
Prematurity;	PROM	4 (10.0)	4 (20.0)	0 (0)	**0.001**
n (%)	Preeclampsia	1 (2.5)	1 (5.0)	0 (0)	
	Average; (range) mean±SD	8.4 ± 0.8 (6 – 9)	8.15 ± 0.9 (6 – 9)	8.6 ± 0.6 (7 – 9)	0.116
APGAR	6 – 7; n (%)	6 (15.0)	5 (25.0)	1 (5.0)	
	8 – 9; n (%)	34 (85.0)	15 (75.0)	19 (95.0)	0.077
Admission cause;	Icterus	20 (50.0)	0 (0)	20 (100)	**<0.001**
n (%)	Sepsis	10 (25.0)	10 (50.0)	0 (0)	
	Formula	7 (17.5)	5 (25.0)	2 (10.0)	
Feeding; n (%)	Breast feeding	20 (50.0)	9 (45.0)	11 (55.0)	0.458
	Both	13 (32.5)	6 (30.0)	7 (35.0)	
Delivery; n (%)	C/Section	38 (95.0)	18 (90.0)	20 (100)	0.487
	NVD	2 (5.0)	2 (10.0)	0 (0)	
WBC count (103/μL)		19.9 ± 7.1 (7.3 – 31.6)	25.9 ± 3.5 (18.0 – 31.6)	13.9 ± 3.9 (7.3 – 20.0)	**<0.001**
Neutrophil count (10	3/μL)	9.9 ± 5.5 (3.5 – 20.0)	14.4 ± 3.9 (7.3 – 20.0)	5.4 ± 2.1 (3.5 – 12.0)	**<0.001**
Platelet count (103/	μL)	293.5 ± 122.5 (127.0 – 623)	262.2 ± 112.6 (127.0 – 623.0)	324.7 ± 126.9 (163.0 – 556.0)	0.108
CRP (mg/L)		8.7 ± 14.1 (1 – 87)	15.5 ± 17.5 (5.0 – 87.0)	1.9 ± 1.3 (1 – 5)	**0.001**
Neonates’ Vitamin	Average; (range) mean±SD μg/dl	19.1 ± 9.4 μg/dl	13.5 ± 3.3 (9.5 – 21.0) μg/dl	24.7 ± 10.2 (11.6 – 54.9) μg/dl	**<0.001**
A status	Deficiency; < 30; n (%)	24 (60.0)	19 (95.0)	5 (25.0)	**<0.001**
	Normal; n ≥ 30 (%)	16 (40.0)	1 (5.0)	15 (75.0)	
	Average; (range) mean±SD	38.4 ± 16.5	30.3 ± 11.4 (15.0 – 50.0) μg/dl	46.5 ± 17.0 (21.0 – 98.4) μg/dl	**0.001**
Mother vitamin A	μg/dl	μg/dl			
	< 30; n (%)	14 (35.0)	11 (55.0)	3 (15.0)	
	≥ 30; n (%)	26 (65.0)	9 (45.0)	17 (85.0)	**0.008**
					

APGAR: Appearance, Pulse, Grimace, Activity, and Respiration; CRP: C-reactive Protein; NVD: Natural Vaginal Delivery; PROM: Premature Rupture of Membranes; RDS: Respiratory Distress Syndrome; SD: Standard Deviation; WBC: white blood cells *Chi-square/Fisher’s exact or independent sample t-test, μg/dl: Microgram per deciliter Bold values indicator of significant association

For a better evaluation of confounding factors, we categorized our septic and non-septic groups based on vitamin A status of the neonates, and evaluated our variables among the two groups. As demonstrated in Table 2, although there was no significant difference among vitamin A levels based on gender, APGAR score, gestational age status, feeding type, and neonatal birth weight, a significant difference regarding vitamin A level was achieved when these factors were compared between the septic and non-septic group (Table 2). Based on a Spearman correlation analysis, there was a significant direct correlation among vitamin A levels in the mothers and the infants (Correlation Coefficient = 0.507; P-value = 0.001).

**Table 2:** Evaluation of neonatal and maternal factors based on vitamin A status among the septic and non-septic neonates.

Variable		Total neonatal Vitamin A	P-value* total	Vitamin A in septic group			Vitamin A in non-septic group			P-value* of average, group
		μg/dl		Average μg/dl	Deficiency; *n* = 19	Normal; *n* = 1	Average μg/dl	Deficiency; *n* = 5	Normal; *n* = 15	
Gender	Female	17.8 ± 10.4 μg/dl	0.428	13.7 ± 3.6 μg/dl	13 (92.9)	1 (7.1)	29.5 ± 14.5 μg/dl	0 (0)	5 (100)	**0.002. 0.001**
	Male	20.2 ± 8.5 μg/dl		13.1 ± 2.7 μg/dl	6 (100)	0 (0)	23.1 ± 8.4 μg/dl	5 (33.3)	10 (66.7)	**0.003, 0.012**
APGAR score	6 – 7	14.9 ± 3.6 μg/dl	0.245	14.1 ± 3.3 μg/dl	5 (100)	0 (0)	19.2 μg/dl	1 (100)	0 (0)	0.143, -
	8 – 9	19.8 ± 9.9 μg/dl		13.3 ± 3.4 μg/dl	14 (93.3)	1 (6.7)	25.0 ± 10.4 μg/dl	4 (21.1)	15 (78.9)	**<0.001, <0.001**
Gestational age	Term	17.4 ± 7.9 μg/dl	0.104	13.5 ± 3.5 μg/dl	14 (93.3)	1 (6.7)	22.3 ± 9.1 μg/dl	8 (66.7)	4 (33.3)	**0.001, 0.003**
	Preterm	22.6 ± 11.6 μg/dl		13.5 ± 3.0 μg/dl	5 (100)	0 (0)	28.2 ± 11.4 μg/dl	1 (12.5)	7 (87.5)	**0.003, 0.005**
Feeding	Formula	15.7 ± 4.9 μg/dl	0.266	13.2 ± 2.7 μg/dl	5 (100)	0 (0)	22.0 ± 2.5 μg/dl	0 (0)	2 (100)	0.053, **0.048**
	Breast feeding	18.1 ± 8.6 μg/dl		13.2 ± 3.5 μg/dl	9 (100)	0 (0)	22.1 ± 9.5 μg/dl	4 (36.4)	7 (63.6)	**0.004, 0.005**
	Both	22.4 ± 11.8 μg/dl		14.2 ± 4.0 μg/dl	5 (83.3)	1 (16.7)	29.4 ± 11.8 μg/dl	1 (14.3)	6 (85.7)	**0.004, 0.029**
Mother vitamin A	< 30	14.5 ± 4.6 μg/dl	**0.022**	12.9 ± 3.2 μg/dl	11 (100)	0 (0)	20.5 ± 4.3 μg/dl	1 (33.3)	2 (66.7)	**0.035, 0.033**
status	≥ 30	21.5 ± 10.4 μg/dl		14.2 ± 3.4 μg/dl	8 (88.9)	1 (11.1)	25.4 ± 10.9 μg/dl	4 (23.5)	13 (76.5)	**0.001, 0.003**
Neonatal birth weight	< 2500	21.8 ± 11.5 μg/dl	0.211	12.0 ± 2.4 μg/dl	3 (27.3)	8 (72.7)	26.1 ± 11.3 μg/dl	2 (22.2)	7 (77.8)	**0.005, 0.021**
	≥ 2500	17.8 μg/± dl 8.1		13.9 μg/± dl 3.5	15 (93.8)	1 (6.3)	23.5 μg/± dl 9.7	4 (100)	0 (0)	**0.002, 0.001**

APGAR: Appearance, Pulse, Grimace, Activity, and Respiration; SD: Standard Deviation; *Chi-square/Fisher’s exact or independent sample t-test/analysis of variance , μg/dl: Microgram per deciliter Bold values indicator of significant association Values are reported as mean ± SD or frequency (%)

Multivariate regression analysis was performed regarding sepsis based on neonatal and maternal factors. Based on our results, only neonates’ vitamin A level had a significant direct association with sepsis (OR: 0.541; P-value=0.017).

## Discussion

Despite it being best to draw conclusions with caution due to the small size of our study population, we found that vitamin A levels were considerably lower in neonates with LOS compared to healthy controls in our investigation, implying that vitamin A insufficiency may play a role in LOS. We also demonstrated that maternal vitamin A levels are correlated with neonatal vitamin A levels. In fact, we had an overwhelming majority of neonates deficient in vitamin A in the LOS group (95% of neonates in LOS group had vitamin A level less than 30 μg/dl).

In recent decades, a large body of research has found evidence of a link between vitamin A deficiency and childhood mortality [23, 24, 25]. Children who had not received vitamin A capsules in the previous 6 months were more likely to be infected, according to a large study conducted in Indonesia [26]. A prospective study of approximately 3000 children found that children with vitamin A deficiency had a 2.17-fold higher risk of enteric infections and a 2.36-fold higher risk of respiratory infections than children with normal vitamin A status [27]. Prior research revealed that the majority of children with hand, foot, and mouth disease had vitamin A insufficiency, which was linked to lower immunity and higher illness severity [16]. Notably, the majority of these studies were conducted in Africa and Southeast Asia, implying a significant prevalence of vitamin A deficiency in these regions, as evidenced by the WHO Global Database on vitamin A deficiency [18].

Sepsis is a biphasic disease; the initial phase is characterized by overwhelming inflammation followed by immuno-suppression, which can lead to multiple organ failure and death. Furthermore, infection among pediatrics could lead to critical consequences. In this investigation, vitamin A deficiency was found to be related to septic shock and severe sepsis on its own. Because vitamin A balances pro- and anti-inflammatory immunity, it’s possible that vitamin A deficiency contributes to the initial septic inflammatory responses. Vitamin A increases the expression and phosphorylation of Smad3 and the expression of Forkhead Box Protein 3 (FOXP3) in anti-inflammatory regulatory T cells, while suppressing the IL-6-induced induction of proinflammatory TH17 cells [28, 29]. Vitamin A has been shown to have a dose-dependent antagonistic effect on IL-6, a protein that plays a key role in development of the systemic inflammatory response syndrome [28, 30]. The inflammation response was worsened in the presence of vitamin A deficiency, which is an undesirable condition for patients with sepsis in the early stages [31], and downregulated inflammatory responses were observed in both human and animal models treated with retinoic acid [32]. Moreover, a prior study found a negative association between vitamin A and CRP levels, indicating that vitamin A deficiency is linked to elevated inflammatory responses [33].

Vitamin A supplementation was first proposed as a way to boost the body’s vitamin A stores in the newborn [34], and recently as a technique to improve infant survival [35]. Three trials, conducted in Indonesia, India, and Bangladesh, have demonstrated that vitamin A supplementation in neonates reduces infant mortality [3, 34, 36]. Lower incidence of LOS in the vitamin A group could be attributed to improved immunological function following vitamin A administration [37]. A non-significant trend toward a reduction in culture-proven nosocomial sepsis was found in a meta-analysis of three published trials [36, 38, 39]. Vitamin A is necessary for optimal cardiovascular development in early pregnancy [40], and it stimulates the development of oxygen-induced ductus arteriosus contraction in the rat model [41], which could explain the much reduced incidence of hemodynamically significant patent ductus arteriosus identified in this study.

Another study found no difference in the spontaneous closure rate of hemodynamically significant patent ductus arteriosus by day 14 after intramuscular vitamin A supplementation (2000–3000 IU/kg IM three times per week for 4 weeks) in ventilator-dependent neonates with very low birth weight [42]. A systematic review and meta-analysis by Rakshasbhuvankar et al. reported that the advantage of vitamin A supplementation for decreasing bronchopulmonary dysplasia is likely to be restricted to infants with a baseline vitamin A intake of 1500 IU per kg per day, and is unaffected by administration mode [43]. Another randomized clinical trial in oxygen dependent infants with GA 32 weeks and birth weight 1250 g found no significant change in the occurrences of RDS, LOS, patent ductus arteriosus, pneumothorax, severe cerebral hemorrhage, retinopathy of prematurity, bronchopulmonary dysplasia, and death with oral vitamin A prophylaxis (30,000 IU/kg for 6 weeks commencing within first 48 h) [44].

There has also been a significant correlation between maternal vitamin A levels and neonatal vitamin A levels. Malama et al. reported that even when infections are the leading cause of death, the findings of their study show that vitamin A supplementation of neonates and postpartum women is unlikely to prevent infant mortality in reasonably well-nourished populations [45]. Whether maternal vitamin A supplements could be beneficial in LOS prevention is still unclear and requires further evaluation.

Regarding the limitations of our study, since it is not possible to design a cohort study and investigate the cause-and-effect relationship between vitamin A deficiency and sepsis due to the high laboratory test cost, we only tried to evaluate the link between sepsis and vitamin A levels. However, matching the case and control groups in terms of other demographic characteristics such as age, sex, weight and gestational age as confounding factors increases the study power and the accuracy of our conclusions. Furthermore, due to the imposed pandemic of COVID-2019 and the unavailability of our patients for routine visits, we were unable to frequently follow up with our patients to evaluate their long-term outcome of vitamin A deficiency and the effects of supplements. Further study in this area would be justified.

In conclusion, our study demonstrated the association of lower levels of vitamin A with an increased risk of LOS, which emphasizes the consideration of vitamin A levels and supplements among neonates and their mothers before and after delivery, because maternal and neonatal vitamin A levels were correlated. This raises the question: Could maternal vitamin A supplementation provide beneficial effects in preventing LOS?

### Discussion on Nutritional Matters

Concerning neonates’ feeding and vitamins intake during the study period, as shown in Table 1, of all 40 neonates in this study, 17.5% were formula fed, 50% were exclusively breastfed, and 32.5% used a combination of formula and breast milk. There was no statistically significant difference between case and control group according to feeding type (p value: 0.4). Also, as it is shown in Table 2, there was no statistically significant difference between vitamin A level in neonates according to their feeding type (p value: 0.26); the reason could be the short duration of neonatal feeding before taking blood samples. Vitamin A level was checked in all neonates between their third and seventh day of life. The average day of admission in these 40 neonates was around the fourth day of their life, which not have been enough time to there to be significant impact on the serum vitamin A level. Based on the statistical results obtained from this study and information shown in Tables 1 and 2, maternal vitamin A status might have a greater effect than the neonatal feeding type on the neonatal vitamin A level and subsequently on the neonatal LOS rate. Regardless of the type of neonatal feeding, serum vitamin A level is significantly lower in the LOS group with any kind of feeding in comparison with the control group with any kind of feeding. Among the factors such as APGAR score, gestational age, birth weight, gender, feeding type and maternal serum vitamin A level, the only factors which were significantly different between the case and control group were maternal and neonatal serum vitamin A level. As there is no de novo fetal vitamin A synthesis, all vitamin A needed by the embryo is gained from maternal vitamin A storage, so it seems that paying attention to the maternal vitamin A level and storage, as well as maternal nutrition, might have a greater effect on the neonatal vitamin A level and consequently on the neonatal LOS rate [48].

Regarding taking vitamin A supplement drops, the average age of neonates who participated in our study was four days. Many infants in our country start taking supplement drops after the first few days of their life. As we mentioned in the exclusion criteria, we excluded any neonates who took supplement drops prior to hospitalization. After hospitalizing both groups of neonates, we also postponed prescribing vitamin A supplementation to the time after we took blood samples to check serum vitamin A level, which was on the first day of their admission.

Regarding using Total Parenteral Nutrition, according to our NICU ward protocol, we start TPN for all neonates as soon as they are admitted if their oral intake was not completely full. Our available TPN solution consists of intralipids, aminofusion and soluvit, none of which include any form of vitamin A. Unfortunately, appropriate intravenous vitamin A solution is not available in our ward. As a result, TPN and nutritional matters are not considered confounding factors in our study.

### Abbreviations

APGAR: Appearance, Pulse, Grimace, Activity, and Respiration; CRP: C-reactive Protein; LOS: Late onset sepsis; NICU: Neonatal intensive care unit; NVD: Natural Vaginal Delivery; PROM: Premature Rupture of Membranes; RDS: Respiratory Distress Syndrome; SD: Standard Deviation; WBC: white blood cells

## Declarations

### Ethics approval and consent to participate

This manuscript has been read and approved by all authors. All protocols were approved by the Ethics Committee of the University. Also, the study was carried out in compliance with the relative guidelines. The Ethics Committee Approval code for this research is IR.IUMS.FMD.REC.1399.576. We ensure quality and integrity of our research. We obtained informed consent from the parents of all infants participating in our study and also obtained informed consent from their mothers who participated in our study. We respect the confidentiality and anonymity of our research respondents. We guarantee that the infants participated in the study voluntarily and according to their parents’ wishes. We also avoided any harm to our participants. There is no conflict of interest in this study, and we did not have any financial support for this research.

## Contributor roles:

Choobdar FA: Conceptualization and Methodology Ghassemzadeh M: writing original draft, editing, and corresponding

Author, Attarian M: Data gathering

Robatmeili L and Aslanbeigi F: Data curation and investigation Rahimian H: Validitation and Visualization

Haghighi B: Supervision

Manafi A: Formal analysis and supervision
